# Human and equipment resources for difficult airway management, airway education programs, and capnometry use in Japanese emergency departments: a nationwide cross-sectional study

**DOI:** 10.1186/s12245-017-0155-6

**Published:** 2017-09-13

**Authors:** Yuko Ono, Koichi Tanigawa, Kazuaki Shinohara, Tetsuhiro Yano, Kotaro Sorimachi, Ryota Inokuchi, Jiro Shimada

**Affiliations:** 1 Emergency and Critical Care Medical Center, Fukushima Medical University Hospital, Fukushima, 960-1295 Japan; 2 Department of Anesthesiology, Ohta General Hospital Foundation, Ohta Nishinouchi Hospital, Koriyama, Japan; 3 Fukushima Global Medical Science Center, Fukushima Medical University, Fukushima, Japan; 4 Department of General and Emergency Medicine, JR Tokyo General Hospital, Tokyo, Japan

**Keywords:** Airway equipment, Capnometry, Supraglottic airway device, Portable storage unit, Postal survey

## Abstract

**Background:**

Although human and equipment resources, proper training, and the verification of endotracheal intubation are vital elements of difficult airway management (DAM), their availability in Japanese emergency departments (EDs) has not been determined. How ED type and patient volume affect DAM preparation is also unclear. We conducted the present survey to address this knowledge gaps.

**Methods:**

This nationwide cross-sectional study was conducted from April to September 2016. All EDs received a mailed questionnaire regarding their DAM resources, airway training methods, and capnometry use for tube placement. Outcome measures were the availability of: (1) 24-h in-house back-up; (2) key DAM resources, including a supraglottic airway device (SGA), a dedicated DAM cart, surgical airway devices, and neuromuscular blocking agents; (3) anesthesiology rotation as part of an airway training program; and (4) the routine use of capnometry to verify tube placement. EDs were classified as academic, tertiary, high-volume (upper quartile of annual ambulance visits), and urban.

**Results:**

Of the 530 EDs, 324 (61.1%) returned completed questionnaires. The availability of in-house back-up coverage, surgical airway devices, and neuromuscular blocking agents was 69.4, 95.7, and 68.5%, respectively. SGAs and dedicated DAM carts were present in 51.5 and 49.7% of the EDs. The rates of routine capnometry use (47.8%) and the availability of an anesthesiology rotation (38.6%) were low. The availability of 24-h back-up coverage was significantly higher in academic EDs and tertiary EDs in both the crude and adjusted analysis. Similarly, neuromuscular blocking agents were more likely to be present in academic EDs, high-volume EDs, and tertiary EDs; and the rate of routine use of capnometry was significantly higher in tertiary EDs in both the crude and adjusted analysis.

**Conclusions:**

In Japanese EDs, the rates of both the availability of SGAs and DAM carts and the use of routine capnometry to confirm tube placement were approximately 50%. These data demonstrate the lack of standard operating procedures for rescue ventilation and post-intubation care. Academic, tertiary, and high-volume EDs were likely to be well prepared for DAM.

**Electronic supplementary material:**

The online version of this article (10.1186/s12245-017-0155-6) contains supplementary material, which is available to authorized users.

## Background

Endotracheal intubation (ETI) is a common and, in many cases, life-saving intervention in emergency departments (EDs). ETI in the ED setting is much more difficult than elective ETI in the operating room (OR), because of the more critical patient population, the lesser controlled setting, and the inadequate opportunity for a complete evaluation of the patient [1, 2]. The rate of difficult ETI in ED settings ranges from 6.1 to 23.5% [1, 3–7], while in planned anesthesia settings it is 0.5–8.5% [8–13]. Consequently, life-threatening ETI-related complications, including hypoxia, esophageal intubation, aspiration, and cardiac arrest, are more likely to occur in the ED [3–5]. These fatal airway-related adverse events can in part be attributed to the limited accessibility of proper human and difficult airway management (DAM) equipment resources [14–17]. Every ED should therefore have the appropriate human and equipment resources for DAM. However, little is known about the availability of either one in Japan’s EDs.

Previous studies [14–17] strongly recommended that, regardless of the location, DAM resources should be consistent with those specified for hospital ORs by several professional anesthesiology societies [18–20]. We previously audited Japanese helicopter physician delivery services [21] and intensive care units (ICU) [22] regarding the adequacy of their equipment and its compliance with DAM guidelines [18–20]. However, whether airway management resources in Japanese EDs are compatible with established OR standards has not been comprehensively evaluated.

In Japan, residency programs in emergency medicine are not standardized [23], and the quality of emergency airway management education depends on the individual institution. Although adequate training in and familiarity with airway management are among the most important elements in emergency medicine [23], objective information on the teaching of airway management in Japanese EDs is not available.

The verification of endotracheal tube placement is an indispensable part of any DAM strategy [18–20], with end-tidal CO_2_ (EtCO_2_) detection as the most accurate method to verify correct tube placement in emergency settings [24–26]. For this reason, secondary ETI confirmation using capnometry is strongly recommended in every ED [14]; however, the level of capnometry use for this purpose in ED patients in Japan is unknown.

Furthermore, there are few data on how ED characteristics and volume affect preparedness for DAM. A consensus regarding this relationship is needed to assess DAM practice variations in each type of ED.

We conducted a national survey to determine: (1) the adequacy of available DAM resources, airway education programs, and post-intubation care, and (2) the association between these DAM preparations and ED characteristics in Japan.

## Methods

### Study design and sites

This cross-sectional study was conducted from April to September 2016 (planning phase, April–June; survey phase, July–September). After its approval (no. 2751) by the Institutional Review Boards of Fukushima Medical University in June 2016, self-administered questionnaires were mailed in July 2016 to the directors of all EDs (530 hospitals in 47 prefectures) registered as certified training facilities by the Japanese Association of Acute Medicine (JAAM). Pre-paid return envelopes with pre-printed addresses were used to increase the response rate, but no incentives were offered. A complete list of these hospitals is available at the official website of the JAAM [27]. The criteria for a JAAM-certified ED include (1) the existence of the facility as an independent, central clinical division; (2) its receipt of a sufficiently large volume of ambulances, patients with cardiopulmonary arrest, and acute-phase patients; (3) two or more dedicated JAAM board-certified ED physicians on staff; and (4) suitable resources and a program for the training of senior residents. EDs that did not respond to the initial survey were sent a repeat mailing in September 2016. No other non-response follow-up techniques, such as phone calls, were used.

### Survey items

Our selection of items for inclusion in the questionnaire was based on previous work in which we investigated available DAM resources in the pre-hospital [21] and ICU [22] settings in Japan. We also referred to all relevant studies conducted in other countries that similarly assessed EDs [28–36], ICUs [37–41], ORs [42–45], and pre-hospital settings [46–48]. We then circulated drafts among the survey team members (an epidemiologist, anesthesiologists, and physicians specializing in emergency medicine) and finalized the questionnaire in April 2016. An English version of the Japanese questionnaire used in this study is available in the Additional file 1 (Online Resource 1). Survey items consisted of facility characteristics, human resources and DAM equipment, airway management training programs, and capnometry use.

#### Facility characteristics

The survey first asked basic information regarding the number of hospital beds and annual ambulance admissions in 2015. EDs were classified as (a) academic or community, (b) high-volume or not, (c) tertiary or not, and (d) urban or suburban and rural. Academic EDs were defined as departments in university-affiliated hospitals, and high-volume EDs were defined as departments in the upper quartile of annual ambulance visits. The criteria for tertiary EDs [49] included (1) 24-h availability of acute care in multiple specialties; (2) the existence of an ICU or coronary care unit that receives critically ill patients; (3) provision of emergency medicine education programs for medical students, junior and senior residents, nurses, and paramedics; and (4) service as a referral medical center for regional emergency medical control. A complete list of Japanese tertiary EDs [50] are available online. The criteria for pediatric EDs were [51]: (1) 24-h availability of care in multiple specialties for critically ill children, (2) a referral resource for communities in nearby regions, (3) provision of continuing education programs in pediatric emergency medicine, and (4) incorporation of a comprehensive quality assessment program. Tertiary and pediatric EDs were both certified by the Japanese Ministry of Health, Labor and Welfare. The census grouping [52] by the Statistics Bureau of the Japanese Ministry of Internal Affairs and Communications was used to identify urban EDs. In brief, urban municipalities included 23 wards within the Tokyo metropolis and 20 ordinance-designated cites. In this study, the EDs were divided into urban and others, with the latter including suburban and rural types.

Of 530 eligible EDs in this survey, 107 (20.2%) were academic, 265 (50%) were tertiary, 185 (34.9%) were urban, and 12 (2.3%) were pediatric EDs.

#### Human resources and DAM equipment

To obtain information on the human resources for airway management, questions were asked about the usual number of on-duty staff ED physician(s) during the day and overnight, the board certification of ED physicians, and whether in-house, experienced (anesthesiology or intensive care medicine) back-up coverage can be called during overnight hours. Senior residents (post-graduate year 3 or more) were defined as staff ED physicians, but junior residents (post-graduate year 1 or 2) were not. “24-h in-house back-up coverage” was deemed obtainable if: (a) two or more physicians were usually on duty, including overnight, or (b) in-house experienced back-up coverage (anesthesiology or intensive care medicine) was available overnight, as previously described [22]. Board-certified physicians were defined based on the Japanese Medical Specialty Board criteria [53].

Equipment resources were queried based on the availability of the following materials in the ED: (1) direct laryngoscope and adjunct equipment (curved blade, straight blade, McCoy laryngoscope, stylet, and gum elastic bougie); (2) alternate intubation equipment (rigid video laryngoscope, flexible fiberscope, retrograde intubation kit, and surgical airway equipment); (3) alternate ventilation equipment [supraglottic airway device (SGA), oral and nasal airways]; (4) a portable packaged unit containing several DAM devices (DAM cart); and (5) analgesics, sedatives, and neuromuscular blocking agents to facilitate ETI, and reversal agents. If a rigid video laryngoscope or SGA was available, respondents were requested to provide the product name. In our previous study [22], SGA availability in Japanese ICUs was determined to be poor, but the reasons were not identified. Thus, in the current survey participants were queried regarding the reasons for the lack of SGA devices in the ED. Surgical airway equipment was categorized as a cricothyroidotomy kit or a set containing a scalpel and hemostat. If a dedicated DAM cart was present in the ED, respondents were asked to specify its contents.

#### Airway management training programs

Emergency medicine residency programs, including DAM educational offerings, vary in length because of the absence of bodies responsible for the accreditation of graduate medical training programs in Japan [23]. To clarify the current situation and to provide a reference point, this survey requested information on the airway management training programs available in each ED, including anesthesiology rotation, DAM simulation training, didactic DAM lecture, and surgical airway training using a simulator, an animal model, a cadaver, etc.

#### Capnometry use

Finally, to determine the current status of capnometry use, both the availability of capnometry (quantitative, colorimetric, or both) in the ED and the extent of capnometry use to confirm tube placement (routinely, sometimes, never) were queried. Our previous study [22] showed that the extent of capnometry use for ETI verification in Japanese ICUs is poor, but the reasons were not explored. Thus, in the present study, respondents were requested to provide reasons for the lack of routine capnometry use to confirm ETI.

### Exposures and outcome measures

The exposures in this study were ED characteristics, including academic, high-volume, tertiary, and urban. Several of these factors were chosen as exposures because previous studies have shown that such hospital characteristics can affect patient outcomes [54–57]. Based on these earlier observations, we hypothesized that such ED types also may be associated with DAM preparedness, airway education, and standardized post-intubation care.

Outcomes of interests in this study were the availability of: (1) 24-h in-house back-up coverage; (2) DAM resources, including (a) SGA, (b) DAM cart, (c) surgical airway equipment, and (d) at least one neuromuscular blocking agent; (3) anesthesiology rotation as an airway management training program; and (4) the routine use of capnometry to confirm ETI. We chose “24-h in-house back-up coverage” as an outcome measure because the “call for help” is the first step and the most important component of DAM algorithms [18–20]. Among the selected DAM equipment, SGA, DAM cart, and surgical airway equipment are commonly endorsed by professional anesthesiology societies [18–20]. The availability of “surgical airway equipment” was defined as the presence in the ED of a cricothyroidotomy kit or a scalpel and hemostat. “Availability of at least one neuromuscular blocking agent” was chosen because the current use of rapid sequence intubation (RSI) in Japanese EDs has yet to be assessed. “Anesthesiology rotation as an airway management training program” is an outcome of interest because of the established association of prior OR exposure with a higher ETI success rate and a lower ETI complication rate in high-risk populations [58–60]. Since post-intubation care with EtCO_2_ detection is strongly recommended following emergency ETI [14, 24–26], the routine use of capnometry for tube placement was included as an outcome measure.

### Statistical analysis

All survey items were evaluated using descriptive statistics. The associations between outcome of interest and ED type (academic, high-volume, tertiary, and urban) were analyzed using a Fisher’s exact test that included only the complete data sets; those with missing data were excluded. Because these four exposures may have overlapped and become confounded by one another, a logistic regression model was constructed to yield an adjusted odds ratio for appropriate DAM preparedness. In this multivariate analysis, a variance-inflation factor was used to detect multicollinearity, and the model’s fit was verified using the Hosmer–Lemeshow goodness-of-fit test. All statistical analyses were performed using IBM SPSS Statistics for Windows, version 21.0 (IBM Corp., Armonk, NY). *P* < 0.05 was considered to indicate statistical significance.

### Sample size

A power analysis using G*Power 3 for Windows (Heinrich Heine University, Dusseldorf, Germany) was performed during the planning phase of this study. The effect size was estimated by referring to our previous work, which determined the association between the ICU type and DAM resources [22]. Based on the assumption that 60% of the EDs had an SGA, DAM cart, and routine use of capnometry for ETI confirmation, the estimated effect size “w” to detect outcome differences of approximately 10% was 0.25. With this effect size, a sample size of 126 per group (total, 252) was calculated to provide 80% statistical power at a two-tailed α of 0.05.

## Results

Of the 530 Japanese EDs, 324 returned a completed questionnaire (response rate 61.1%). Table 1 shows the facility characteristics of the responding EDs. The median number of annual ambulances admissions was 4044 (interquartile range 2838–5728). Of these, 24.4% were academic EDs and 56.8% tertiary EDs.

**Table 1:** Demographic data of the Japanese emergency departments (EDs) that responded to the survey^a^

Basic information	Median (inter-quartile range)
Hospital beds	507 (390–684)
Annual ED visits by ambulance	4044 (2838–5728)
ED type	*N* (%)
By funding institute (*N* = 324)	
Academic^b^	79 (24.4)
Community	245 (75.6)
By volume (*N* = 319)^c^	
High-volume^d^	80 (25.1)
Other	239 (74.9)
By management level (*N* = 324)	
Tertiary^e^	184 (56.8)
Secondary or primary	140 (43.2)
By location (*N* = 324)	
Urban^f^	117 (36.1)
Suburban or rural	207 (63.9)
By specialty (*N* = 324)	
Pediatric^g^	8 (2.5)
Other	316 (97.5)

^a^Based on the replies of 324 of the 530 EDs queried

^b^Defined as EDs in university-affiliated hospitals

^c^There were five missing data

^d^Defined as EDs in the upper quartile of annual ambulance visits (> 5728)

^e^Defined as EDs in referral medical centers of regional emergency medical control that are certified by the Japanese Ministry of Health, Labor and Welfare

^f^Defined using the census grouping criteria by the Statistics Bureau of the Japanese Ministry of Internal Affairs and Communications

^g^Defined as EDs with a referral resource for critically ill children for communities in nearby regions that are certified by the Japanese Ministry of Health, Labor and Welfare

Table 2 provides data on ED manpower and the specialties of the ED physicians. Two or more staff members were usually on duty at 76.3% of the responding EDs during the day, and at 55.2% overnight. In-house back-up coverage was always available in 69.4% of the EDs. In Japan, other than physicians specialized in emergency medicine, those from various specialties, including general surgery, cardiovascular medicine, intensive care, and anesthesiology, serve as ED practitioners (Table 2).

**Table 2:** Number of on-duty emergency department (ED) physicians and their specialties^a^

Item	*N* (%)
Number of on-duty ED physicians	317^b^
A. Day time	
a) One	75 (23.7)
b) Two or more	242 (76.3)
B. Overnight	
a) One	142 (44.8)
b) Two or more	175 (55.2)
c) In-house back-up coverage^c^ always available	220 (69.4)
Board certification of ED physicians^d^	*N* = 3697
a) Emergency medicine	1223 (33.1)
b) General surgery	726 (19.6)
c) Cardiovascular medicine	350 (9.5)
d) Orthopedics	328 (8.9)
e) Anesthesiology	322 (8.7)
f) Intensive care	313 (8.5)
g) Cranial surgery	266 (7.2)
h) Pediatrics	202 (5.5)
i) Respiratory medicine	126 (3.4)
j) Renal medicine	88 (2.4)
k) Cardiovascular surgery	78 (2.1)
l) Other board certification	579 (15.7)

^a^Based on the replies of 324 of the 530 EDs queried

^b^There were seven missing replies

^c^Two or more ED physicians are always on duty or in-house experienced back-up coverage (anesthesiology or intensive care medicine) is usually available overnight

^d^Physicians may have more than one board certification

Table 3 summarizes the intubation and alternate intubation equipment available in Japanese EDs. Among the EDs that responded, a curved laryngoscope blade was universally available, and nearly all EDs (*n* = 310, 95.7%) possessed a surgical airway device, either a cricothyroidotomy kit (75.9%) or scalpel and hemostat (19.8%).

**Table 3:** Intubation equipment and alternate intubation equipment in the Japanese emergency departments (EDs) that responded to the survey^a^

Equipment item	*N* (%)
Direct laryngoscope and adjunct equipment^b^	
Curved laryngoscope blade (Macintosh type)	324 (100)
Assorted sizes	319 (98.5)
Straight laryngoscope blade (Miller type)	179 (55.2)
Assorted sizes	159 (49.1)
McCoy laryngoscope	55 (17.0)
Stylet	321 (99.1)
Gum elastic bougie	159 (49.1)
Alternate intubation equipment	
Rigid video laryngoscope^b^	285 (88.0)
Airway scope®	228 (70.4)
McGRATH MAC®	162 (50.0)
GlideScope®	11 (3.4)
C-MAC®	10 (3.1)
King Vision®	6 (1.9)
Other	8 (2.5)
Flexible fiberscope	195 (60.2)
Retrograde intubation kit	9 (2.8)
Surgical airway equipment	310 (95.7)
Cricothyroidotomy kit	246 (75.9)
Only scalpel and hemostat	64 (19.8)

^a^Based on the replies of 324 of the 530 EDs queried

^b^EDs may have more than one of the specified equipment items

Table 4 lists the available alternate ventilation equipment in the responding EDs. SGA availability was 51.5%. The performance of a surgical airway in patients with difficult ETI (58.6%) and a lack of familiarity with SGA insertion (39.5%) were the main reasons for the lack of a SGA in the ED.

**Table 4:** Alternate ventilation equipment in responded Japanese emergency departments (EDs)^a^

Equipment item	*N* (%)
Alternate ventilation equipment^b^	
Oral airway	278 (85.8)
Nasal airway	313 (96.6)
SGA^b^	167 (51.5)
I-gel®	102 (31.5)
LMA Classic®	39 (12.0)
LMA ProSeal®	28 (8.6)
Air-Q®	11 (3.4)
Laryngeal tube®	6 (1.9)
LMA Fastrach®	2 (0.6)
LMA Supreme®	2 (0.6)
Others	6 (1.9)
Reason for lack of SGA^c^	*N* = 157
A surgical airway is performed if ETI is difficult	92 (58.6)
Lack of familiarity	62 (39.5)
Perceived as not useful for emergency cases	29 (18.5)
Expensive	5 (3.2)
Other	38 (24.2)

*SGA* supraglottic airway device

^a^Based on the replies of 324 of the 530 EDs queried

^b^EDs may have more than one of the specified equipment items

^c^EDs may have more than one reason

Dedicated DAM carts were present in 161 (49.7%) EDs and their contents varied (Table 5).

**Table 5:** Portable storage unit (DAM cart) and its contents available at the responding Japanese emergency departments (EDs)^a^

Item	*N* (%)
Portable storage unit (DAM cart)	161 (49.7)
Contents of the DAM cart	161
Stylet	145 (90.1)
Direct laryngoscope blades in various designs and sizes	142 (88.2)
Tracheal tubes in assorted sizes	135 (83.9)
Magill forceps	129 (80.1)
Airway (oral/nasal)	127 (78.9)
Bag valve mask	122 (75.8)
Rigid video laryngoscope	107 (66.5)
Surgical airway device	100 (62.1)
SGA	68 (42.2)
Gum elastic bougie	67 (41.6)
Capnometry	51 (31.7)
Yankauer suction tip	39 (24.2)
Sugammadex	8 (5.0)
Other devices	8 (5.0)

*DAM* difficult airway management, *SGA* supraglottic airway device

^a^Based on the replies of 324 of the 530 EDs queried

Table 6 lists the drugs available to facilitate ETI in the responding EDs. At least one neuromuscular agent was cited by 222 (68.5%) EDs and at least one opioid by 135 (41.7%) EDs.

**Table 6:** Drugs to facilitate ETI and reversal agents available at the responding Japanese emergency departments (EDs)^a^

Item	*N* (%)
Analgesics^b^	
At least one opioid	135 (41.7)
Fentanyl	116 (35.8)
Morphine	95 (29.3)
Remifentanil	3 (0.9)
Ketamine	77 (23.8)
Pentazocin	278 (85.8)
Buprenorphine	144 (44.4)
Tramadol	3 (0.9)
Lidocaine	251 (77.5)
Other	7 (2.2)
Sedatives^b^	
At least one sedative	324 (100)
Diazepam	300 (92.6)
Midazolam	293 (90.4)
Propofol	237 (73.1)
Thiopental	153 (47.2)
Dexmedetomidine	83 (25.6)
Haloperidol	163 (50.3)
Droperidol	17 (5.2)
Other	3 (0.9)
Neuromuscular blocking agents^b^	
At least one neuromuscular blocking agent	222 (68.5)
Rocuronium	187 (57.7)
Vecuronium	72 (22.2)
Pancuronium	2 (0.6)
Succinylcholine	22 (6.8)
Reversal agents^b^	
Sugammadex	74 (22.8)
Flumazenil	159 (49.1)
Naloxone	50 (15.4)
Neostigmine	38 (11.7)

*ETI* endotracheal intubation

^a^Based on the replies of 324 of the 530 EDs queried

^b^EDs may have more than one drug

Table 7 provides details on the airway teaching programs in Japanese EDs. Diverse DAM training methods are used in the education of ED physicians. An anesthesiology rotation was available in 125 EDs (38.6%).

**Table 7:** Airway management teaching programs available at the responding Japanese emergency departments (EDs)^a^

Airway management teaching program^b^	*N* (%)
Anesthesiology rotation	125 (38.6)
Surgical airway training using a simulator, an animal model, a cadaver, etc.	99 (30.6)
DAM simulation training	56 (17.3)
Didactic lecture	47 (14.5)
Other program	36 (11.1)

*DAM* difficult airway management

^a^Based on the replies of 324 of the 530 EDs queried

^b^EDs may have more than one airway management teaching program

Information regarding post-intubation care with EtCO_2_ detection is provided in Table 8. Despite the high availability of capnometry, its routine use for ETI was reported by less than half (47.8%) of the EDs. The major reasons for not routinely using capnometry to verify tube placement were ETI confirmation by other methods, such as tube fogging, chest rise, direct visualization, and auscultation (52.7%), and that its use depended on the discretion of the ED physician (47.3%).

**Table 8:** Current status regarding capnometry use for ETI among the responding Japanese emergency departments (EDs)^a^

Item	*N* (%)
Capnometry^b^	
Quantitative capnometry	270 (83.3)
Colorimetric capnometry	82 (25.3)
Use of capnometry to confirm ETI	316^c^
Routinely	151 (47.8)
Sometimes	106 (33.5)
Never	59 (18.7)
Reason for lack of routine use of capnometry to confirm ETI	165^d^
Confirmation by other methods (e.g., tube fogging, chest rise, direct visualization, and auscultation)	87 (52.7)
Discretion of ED physicians	78 (47.3)
Expensive	18 (10.9)
Device shortage	16 (9.7)
Lack of familiarity	11 (6.7)
Other	13 (7.9)

*ETI* endotracheal intubation

^a^Based on the replies of 324 of the 530 EDs queried

^b^EDs may have both types of capnometry

^c^There are eight missing data

^d^EDs may have more than one reason

**Fig. 1:**
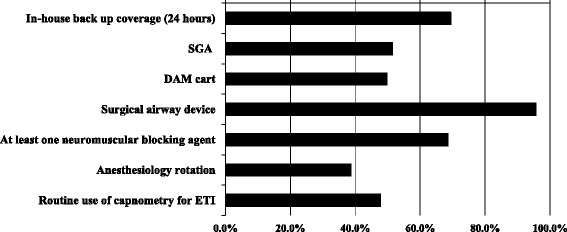
Availability of important difficult airway management (DAM) resources and of a clinical anesthesia rotation, as well as the use of capnometry in Japanese emergency departments. *ETI* endotracheal intubation, *SGA* supraglottic airway device

Figure 1 summarizes the attainment rates of the outcomes of interest in this study. According to our definitions, back-up staff was always available in 69.4% of the EDs, surgical airway devices in 95.7%, and neuromuscular blocking agents in 68.5%. The availability of SGAs and DAM carts, as well as routine capnometry use to confirm tube placement was approximately 50%. The availability of an anesthesiology rotation for ED physicians was low (< 40%).

Table 9 shows the associations between the feasibility of the outcomes of interest and the ED type. The availability of 24-h back-up coverage was significantly higher in academic EDs and tertiary EDs in both the crude and adjusted analysis. Similarly, neuromuscular blocking agents were more likely to be present in academic EDs, high-volume EDs, and tertiary EDs; an anesthesiology rotation was significantly less available in academic EDs; and the rate of routine capnometry use to verify ETI was significantly higher in tertiary EDs in both the crude and adjusted analysis. Multicollinearity was not detected (variance-inflation factor < 1.2 for each explanatory variable of each model), and the Hosmer–Lemeshow test verified the good fit (*P* > 0.05) of each logistic regression model.

**Table 9:** Association between outcomes of interest and emergency department (ED) type

Item	%	Crude analysis		Adjusted analysis	
		Odds ratio (95% CI)	*P*	Odds ratio (95% CI)	*P*
24-h back-up coverage					
Academic ED	84.8	3.4 (1.7–6.5)	< 0.001	3.3 (1.7–6.8)	< 0.001
High-volume ED	73.8	1.4 (0.8–2.5)	0.3	1.4 (0.7–2.5)	0.3
Tertiary ED	73.9	1.9 (1.2–3.0)	0.008	1.8 (1.1–3.0)	0.02
Urban ED	75.2	1.7 (1.1–2.9)	0.03	1.7 (1.0–2.9)	0.06
Supraglottic airway device					
Academic ED	60.8	1.6 (1.0–2.4)	0.07	1.6 (1.0–2.8)	0.08
High-volume ED	56.2	1.3 (0.8–2.2)	0.3	1.4 (0.8–2.4)	0.2
Tertiary ED	54.3	1.3 (0.8–2.0)	0.3	1.2 (0.7–1.8)	0.5
Urban ED	49.6	0.9 (0.6–1.4)	0.6	0.8 (0.5–1.3)	0.4
Surgical airway device					
Academic ED	93.7	0.6 (0.2–1.7)	0.3	0.7 (0.2–2.3)	0.5
High-volume ED	95	0.7 (0.2–2.5)	0.7	0.7 (0.2–2.6)	0.6
Tertiary ED	95.7	1.0 (0.3–2.9)	1	1.2 (0.4–3.7)	0.8
Urban ED	94	0.6 (0.2–1.6)	0.3	0.7 (0.2–2.2)	0.5
DAM cart					
Academic ED	49.4	1.0 (0.6–1.6)	1	1.0 (0.6–1.7)	0.9
High-volume ED	52.5	1.1 (0.7–1.9)	0.7	1.2 (0.7–2.0)	0.6
Tertiary ED	49.5	1.0 (0.6–1.5)	1	1.0 (0.6–1.5)	1
Urban ED	47.9	0.9 (0.6–1.4)	0.6	0.9 (0.6–1.4)	0.6
Neuromuscular blocking agents					
Academic ED	83.5	2.9 (1.5–5.5)	0.001	3.2 (1.6–6.5)	0.001
High-volume ED	80	2.2 (1.2–4.1)	0.01	2.2 (1.1–4.1)	0.02
Tertiary ED	78.8	3.0 (1.9–4.9)	< 0.001	2.7 (1.6–4.4)	< 0.001
Urban ED	68.4	1.0 (0.6–1.6)	1	0.9 (0.5–1.6)	0.9
Anesthesiology rotation					
Academic ED	20.3	0.3 (0.2–0.6)	< 0.001	0.3 (0.2–0.6)	< 0.001
High-volume ED	38.8	1.0 (0.6–1.7)	1	1.0 (0.6–1.7)	0.9
Tertiary ED	33.7	0.6 (0.4–1.0)	0.05	0.7 (0.4–1.1)	0.08
Urban ED	35	0.8 (0.5–1.3)	0.34	0.8 (0.5–1.4)	0.5
Routine use of capnometry to confirm ETI					
Academic ED	53.2	1.4 (0.9–2.4)	0.2	1.3 (0.8–2.3)	0.3
High-volume ED	47.5	1.0 (0.6–1.7)	1	0.9 (0.5–1.6)	0.8
Tertiary ED	54.3	2.1 (1.3–3.3)	0.002	2.1 (1.3–3.3)	0.002
Urban ED	45.3	0.9 (0.6–1.5)	0.7	0.9 (0.6–1.5)	0.7

*CI* confidence interval, *DAM* difficult airway management, *ETI* endotracheal intubation

Academic ED, high-volume ED, and tertiary ED are defined in Table 1

An international comparison of the outcomes of interest in this study is provided in Additional file 2: Table S1.

The differences in characteristics between the respondent and non-respondent EDs were compared to assess non-response bias. As shown in Additional file 3: Table S2, respondent EDs were likely to be academic EDs (*P* = 0.003) and tertiary EDs (*P* < 0.001).

## Discussion

This national survey examined the currently available human, drug, and equipment resources for DAM and the extent of capnometry use in Japanese EDs. Roughly two-thirds of the responding EDs were supplied with neuromuscular blocking agents; in half of the EDs, SGAs and dedicated DAM carts were available and capnometry was routinely used to verify tube placement. These data suggest that airway management practices, including RSI use, performance of a rescue strategy, and post-intubation care, vary in Japanese EDs. This may in part be due to differences in the airway management education offerings. Academic, tertiary, and high-volume EDs were likely to be well prepared for DAM.

Among the responding EDs, SGA was available in only 51.5% (Table 4). Therefore, in Japan, SGA is under-used as a rescue ventilation device. The main reason reported for the limited availability of SGAs is that a surgical airway is typically performed when a difficult airway is encountered (Table 4). Many Japanese ED physicians may choose to perform a definitive surgical airway rather than rescue ventilation through SGA when patient ventilation and/or intubation are difficult. Another important cause contributing to the low-level use of SGAs in Japanese EDs is insufficient familiarity with their placement (Table 4). Appropriate SGA training for ED physicians is limited in Japanese EDs because, other than elective operations, the settings in which patients are ventilated with a SGA are relatively rare and truly emergent. This study also revealed the low availability of an anesthesiology rotation for ED physicians (Table 7). As previously noted [61, 62], training in the hospital OR to gain SGA insertion experience and confidence would be beneficial for many ED practitioners.

A dedicated DAM cart was present in less than half the EDs and its contents varied considerably (Table 5). Because airway difficulties are far more likely in the ED [1, 3–7] and time is very limited in the airway management of a critically ill patient, every ED should have immediate access to at least one DAM cart, which should have the same contents and layout as that used in the respective hospital’s OR [14]. Berkow et al. [63] reported that, after the implementation of a comprehensive airway program, including standardized DAM cart preparation, the need for an emergency surgical airway decreased.

Approximately one-third of the responding EDs were not equipped with neuromuscular blocking agents (Table 6), indicative of the variable use of RSI across Japanese EDs. In their multicenter observational study of 10 academic and community Japanese EDs, Hasegawa et al. [23] observed a high degree of variation in airway management practices among hospitals, with those using RSI accounting for 0–79%. The findings from our cross-sectional study of 324 hospitals support this high degree of variability. We also found a significantly higher availability of neuromuscular blocking agents in academic EDs, high-volume EDs, and tertiary EDs (Table 9). Thus, RSI is more likely to be used in these types of EDs than in community, small-volume, or secondary EDs.

Less than half of the EDs routinely used capnometry for ETI verification (Table 8). The major reasons were the confirmation of ETI by other methods, such as tube fogging and auscultation, and that capnometry use was left to the discretion of the ED physician (Table 8). Thus, standard operating procedures for post-intubation care are lacking in many Japanese EDs. Previous studies [14, 16] showed that the increased use of capnography was the single change with the greatest potential to prevent death from airway complications outside the OR. The further incorporation of ETCO_2_ confirmation in Japanese EDs would thus improve patient outcomes.

The clinical backgrounds of the ED physicians in our study were highly diverse (Table 2). Therefore, in Japanese EDs, there may be varying levels of airway management expertise. O’Malley et al. [64] referred to this diversity as a multispecialty staffing model.

Our data also revealed differences in the methods used in airway management training for emergency medicine trainees, including OR exposure (Table 7). The diversity of the educational offering in airway management may, at least in part, explain the resource and practice variations with respect to RSI, rescue strategy, and post-intubation care. In Japan, airway management education, including quality and quantity endpoints, has not been standardized because of the absence of bodies that accredit the residency program [23]. Our study provides a reference point for DAM education programs available in Japanese EDs and offers the opportunity for the directors of each emergency medicine residency program to reappraise their own education offerings.

Finally, this study found a general trend that academic EDs, high-volume EDs, and tertiary EDs were well prepared in terms of their DAM resources, including 24-h back-up coverage and the availability of neuromuscular blocking agents (Table 9). It also showed that capnometry was more likely to be used for ETI verification in tertiary EDs. Previous studies [54, 55] demonstrated that patient outcomes at this type of ED were better than at other types. These findings collectively suggest that better DAM resources and post-intubation care are associated with improved patient management. We also determined that an anesthesia rotation was far less commonly available at academic EDs (Table 9), suggesting that community EDs were the most likely to have flexible airway rotation programs for ED physicians.

### Study limitations and advantages

Our study had four major limitations. First, the survey did not include non-JAAM-certified EDs, because a complete list of non-JAAM-certified training facilities was not available. However, it is likely that DAM resources are less available and capnometry is used less often in these hospitals because most are not academic EDs, high-volume EDs, or tertiary EDs. Second, the frequencies of difficult airways situations (i.e., cannot ventilate and cannot intubate) were neither determined nor was information obtained on airway management practices in Japanese EDs. Third, because our questionnaire was self-administered, reporting bias was possible. Fourth, as in any study using questionnaires, this study may be affected by non-response bias. Actual DAM resources and post-intubation care using capnometry in JAAM-certified EDs may be even poorer because respondents of this survey were likely to be academic and tertiary EDs (Additional file 3: Table S2).

In spite of these limitations, this study also had several strengths. First, the response rate was relatively high (324 of 530 surveyed EDs), and the survey assessed various types of EDs, including academic, community, tertiary, urban, and pediatric, located in many geographic areas of Japan. Therefore, our data accurately reflect the current status of advanced airway management across the country. Second, our findings are the first to demonstrate associations between ED type, the availability of neuromuscular blocking agents, and the availability of an anesthesia rotation. Overall, our study identified areas in need of improvement regarding DAM resources and post-intubation care. Our survey provides the opportunity for each ED to reappraise its own DAM resources, education, and practice. We believe this quality improvement would be beneficial not only for Japanese EDs but also for EDs in other countries.

## Conclusions

This nationwide cross-sectional study demonstrated wide-ranging differences in airway management resources in Japanese EDs. Neuromuscular blocking agents, SGAs, and DAM carts are of limited availability, while the use of capnometry to confirm correct tube placement is not universal. These data imply that RSI, rescue strategies, and post-intubation care in Japanese ED also vary and are not standardized. Academic, tertiary, and high-volume EDs were likely to be well prepared for DAM. We believe this study is a meaningful first approach to improving DAM resources and practice in Japanese EDs.

## Additional files

Additional file 1: Survey of airway management resources in Japanese emergency departments. (DOCX 26 kb)

Additional file 2: Table S1. International comparison of outcomes of interests with the outcome determined in this study. (DOCX 14 kb)

Additional file 3: Table S2. Characteristic differences between respondent vs. non-respondent emergency departments (EDs). (DOCX 13 kb)

## Abbreviations

CI: Confidence interval
DAM: Difficult airway management
ED: Emergency department
EtCO_2_: End-tidal CO_2_
ETI: Endotracheal intubation
ICU: Intensive care units
JAAM: Japanese Association of Acute Medicine
OR: Operating room
RSI: Rapid sequence intubation
SGA: Supraglottic airway device
