# Supplementary material for: Human and equipment resources for difficult airway management, airway education programs, and capnometry use in Japanese emergency departments: a nationwide cross-sectional study
Source: Int J Emerg Med. 2017 Sep 13;10:28. doi: 10.1186/s12245-017-0155-6 (PMC5597568; doi:10.1186/s12245-017-0155-6)
Supplement: Supplementary file 1 — Survey of airway management resources in Japanese emergency departments. (DOCX 26 kb) [file 12245_2017_155_MOESM1_ESM.docx]

| **Survey of airway management resources in Japanese emergency departments** | | | | | | | | | | | | | | | | | | | | | | | | | | | | | | | | | | | | | | | | | | | | | | | | | | | | | | | | | | | | | | | | | | | | | | | | | | | | | | | | | | | | | | | | | | | | | | | | | | | | | | | | | | | | | |  |  |  |  |  |  |  |
| --- | --- | --- | --- | --- | --- | --- | --- | --- | --- | --- | --- | --- | --- | --- | --- | --- | --- | --- | --- | --- | --- | --- | --- | --- | --- | --- | --- | --- | --- | --- | --- | --- | --- | --- | --- | --- | --- | --- | --- | --- | --- | --- | --- | --- | --- | --- | --- | --- | --- | --- | --- | --- | --- | --- | --- | --- | --- | --- | --- | --- | --- | --- | --- | --- | --- | --- | --- | --- | --- | --- | --- | --- | --- | --- | --- | --- | --- | --- | --- | --- | --- | --- | --- | --- | --- | --- | --- | --- | --- | --- | --- | --- | --- | --- | --- | --- | --- | --- | --- | --- | --- | --- | --- | --- | --- | --- | --- | --- | --- | --- | --- | --- | --- | --- | --- | --- |
|  |  | |  | |  | |  | |  | |  | |  | |  | |  | |  | | |  | |  | |  | |  | |  | | |  | | | | | |  | | | | |  | |  | |  | | | |  | | | | | |  | | | |  | | | |  | | | |  | | | |  | | | |  | | | |  | | | |  | | | |  | | | |  | | | |  | | | | |  | | | |  | | | |  | |  | |  | |
|  |  | |  | | **Note**  Please fill out this form based on the situation in your hospital in July 2016, unless any annotations are provided. | | | | | | | | | | | | | | | | | | | | | | | | | | | | | | | | | | | | | | | | | | | | | | | | | | | | | | | | | | | | | | | | | | | | | | | | | | | | | | | | | | | | | | | | | | | | | |  | | | | |  | | | | | |  |  |  |  |  |  |  |
|  |  | |  | |  |  |  |  |  |  |  |  |  |  |  |  |  |  |  |  |  |  |  |  |  |  |  |  |  |  |  |  |  |  |  |  |  |  |  |  |  |  |  |  |  |  |  |  |  |  |  |  |  |  |  |  |  |  |  |  |  |  |  |  |  |  |  |  |  |  |  |  |  |  |  |  |  |  |  |  |  |  |  |  |  |  |  |  |  |  |  |  |  |  |  |  |  |  |  | | | | |  | | | | | |  |  |  |  |  |  |  |
|  |  | |  | |  |  |  |  |  |  |  |  |  |  |  |  |  |  |  |  |  |  |  |  |  |  |  |  |  |  |  |  |  |  |  |  |  |  |  |  |  |  |  |  |  |  |  |  |  |  |  |  |  |  |  |  |  |  |  |  |  |  |  |  |  |  |  |  |  |  |  |  |  |  |  |  |  |  |  |  |  |  |  |  |  |  |  |  |  |  |  |  |  |  |  |  |  |  |  | | | | |  | | | | | |  |  |  |  |  |  |  |
|  |  | |  | |  | |  | |  | |  | |  | |  | |  | |  | | |  | |  | |  | |  | |  | | |  | | | | | |  | | | | |  | |  | |  | | | |  | | | | | |  | | | |  | | | |  | | | |  | | | |  | | | |  | | | |  | | | |  | | | |  | | | |  | | | |  | | | | |  | | | |  | | | |  | |  | |  | |
| 1. **Hospital name** | | | | | | | | | | | | | | | | | | | | | | | | | | | | | | | | | | | |  | | | | | | | | | | | | | | | | | | | | | | | | | | | | | | | | | | | | | | | | | | | | | | | | | | | | | | | | | | | | | | | | | | | | | | | | | |  |  |  |  |  |  |  |
| 1. **How many beds does your hospital have?** | | | | | | | | | | | | | | | | | | | | | | | | | | | | | | | | | | | |  | | | | | | | | | | | | | | | | | | | | | | | | | | | | | | | | | | | | | | | | | | | | | | | | | | | | | | | | | | | | | | | | | | | | | | | | | |  |  |  |  |  |  |  |
| 1. **How many ambulances did your emergency department (ED) receive in 2015?** | | | | | | | | | | | | | | | | | | | | | | | | | | | | | | | | | | | |  | | | | | | | | | | | | | | | | | | | | | | | | | | | | | | | | | | | | | | | | | | | | | | | | | | | | | | | | | | | | | | | | | | | | | | | | | |  |  |  |  |  |  |  |
| 1. **Is the following airway equipment available in your ED?** | | | | | | | | | | | | | | | | | | | | | | | | | | | | | | | | | | | | | | | | | | | | | | | | | | | | | | | | | | | | | | | | | | | | | | | | | | | | | | | | | | | | | | | | | | | | | | | | | | | | | | | | | | | | | |  |  |  |  |  |  |  |
| 1. Direct laryngoscope and adjunct equipment | | | | | | | | | | | | | | | | | | | | | | | | | | | | | | | | | | | |  | |  | | | |  | | | | | | |  | | | |  | | |  | | | |  | | | |  | | | |  | | | |  | | | |  | | | |  | | | |  | | | |  | | | |  | | | |  | | | | |  | | | | |  | | | |  |  |  |  |  |  |  |
| *A.* Curved laryngoscope blade (Macintosh type)  *(a)* assorted sizes | | | | | | | | | | | | | | | | | | | | | | | | | | | | | | | | | | | | | | | | **□ Yes □ No**  **□ Yes □ No** | | | | | | | | | | | | | | | | | | | | | | | | | | | | | | | | | | | | | | | | | | | | | | | | | | | | | | | | | | | | | | | | | | | | | |  |  |  |  |  |  |  |
| *B.* Straight laryngoscope blade (Miller type)  *(a)* Assorted sizes | | | | | | | | | | | | | | | | | | | | | | | | | | | | | | | | | | | | | | | | **□ Yes □ No**  **□ Yes □ No** | | | | | | | | | | | | | | | | | | | | | | | | | | | | | | | | | | | | | | | | | | | | | | | | | | | | | | | | | | | | | | | | | | | | | |  |  |  |  |  |  |  |
| *C.* McCoy laryngoscope | | | | | | | | | | | | | | | | | | | | | | | | | | | | | | | | | | | | | | | | **□ Yes □ No** | | | | | | | | | | | | | | | | | | | | | | | | | | | | | | | | | | | | | | | | | | | | | | | | | | | | | | | | | | | | | | | | | | | | | |  |  |  |  |  |  |  |
| *D.* Stylet | | | | | | | | | | | | | | | | | | | | | | | | | | | | | | | | | | | | | | | | **□ Yes □ No** | | | | | | | | | | | | | | | | | | | | | | | | | | | | | | | | | | | | | | | | | | | | | | | | | | | | | | | | | | | | | | | | | | | | | |  |  |  |  |  |  |  |
| *E.* Gum elastic bougie | | | | | | | | | | | | | | | | | | | | | | | | | | | | | | | | | | | | | | | | **□ Yes □ No** | | | | | | | | | | | | | | | | | | | | | | | | | | | | | | | | | | | | | | | | | | | | | | | | | | | | | | | | | | | | | | | | | | | | | |  |  |  |  |  |  |  |
| (2) Alternative intubation equipment | | | | | | | | | | | | | | | | | | | | | | | | | | | | | | | | | | | | | | | | | | | | | | | | | | | | | | | | | | | | | | | | | | | | | | | | | | | | | | | | | | | | | | | | | | | | | | | | | | | | | | | | | | | | | |  |  |  |  |  |  |  |
| *A.* Rigid video laryngoscope | | | | | | | | | | | | | | | | | | | | | | | | | | | | | | | | | | | | | | | | **□ Yes □ No** | | | | | | | | | | | | | | | | | | | | | | | | | | | | | | | | | | | | | | | | | | | | | | | | | | | | | | | | | | | | | | | | | | | | | |  |  |  |  |  |  |  |
| *B.* If yes, please provide the product name.  If your ED has more than one rigid video laryngoscope, please enumerate. | | | | | | | | | | | | | | | | | | | | | | | | | | | | | | | | | | | | | | | |  | | | | | | | | | | | | | | | | | | | | | | | | | | | | | | | | | | | | | | | | | | | | | | | | | | | | | | | | | | | | | | | | | | | | | |  |  |  |  |  |  |  |
| *C.* Flexible fiberscope | | | | | | | | | | | | | | | | | | | | | | | | | | | | | | | | | | | | | | | | **□ Yes □ No** | | | | | | | | | | | | | | | | | | | | | | | | | | | | | | | | | | | | | | | | | | | | | | | | | | | | | | | | | | | | | | | | | | | | | |  |  |  |  |  |  |  |
| *D.* Retrograde intubation kit | | | | | | | | | | | | | | | | | | | | | | | | | | | | | | | | | | | | | | | | **□ Yes □ No** | | | | | | | | | | | | | | | | | | | | | | | | | | | | | | | | | | | | | | | | | | | | | | | | | | | | | | | | | | | | | | | | | | | | | |  |  |  |  |  |  |  |
| (3) Alternative ventilation equipment | | | | | | | | | | | | | | | | | | | | | | | | | | | | | | | | | | | | | | | | |  | | | | | |  | | |  | | | | |  | |  | | | |  | | | |  | | | |  | | | |  | | | |  | | | |  | | | |  | | | |  | | | |  | | | |  | | | | |  | | | | | | |  |  |  |  |  |  |  |  |
| *A.* Supraglottic airway device (SGA) | | | | | | | | | | | | | | | | | | | | | | | | | | | | | | | | | | | | | | | | | **□ Yes □ No**  **□ Yes □ No** | | | | | | | | | | | | | | | | | | | | | | | | | | | | | | | | | | | | | | | | | | | | | | | | | | | | | | | | | | | | | | | | | | | |  |  |  |  |  |  |  |  |
| *(a)* Assorted sizes | | | | | | | | | | | | | | | | | | | | | | | | | | | | | | | | | | | | | | | | |  |  |  |  |  |  |  |  |  |  |  |  |  |  |  |  |  |  |  |  |  |  |  |  |  |  |  |  |  |  |  |  |  |  |  |  |  |  |  |  |  |  |  |  |  |  |  |  |  |  |  |  |  |  |  |  |  |  |  |  |  |  |  |  |  |  |  |  |  |  |  |  |  |  |  |  |
| *B.* If you answered ***Yes*** to ***A***, please provide the product name. If your ED has more than one SGA, please enumerate. | | | | | | | | | | | | | | | | | | | | | | | | | | | | | | | | | | | | | | | | |  | | | | | | | | | | | | | | | | | | | | | | | | | | | | | | | | | | | | | | | | | | | | | | | | | | | | | | | | | | | | | | | | | | | |  |  |  |  |  |  |  |  |
| *C.* If you answered ***No*** to ***A***, what are the reasons? (Please select all that apply.) | | | | | | | | | | | | | | | | | | | | | | | | | | | | | | | | | | | | | | | | | | | | | | | | | | | | | | | | | | | | | | | | | | | | | | | | | | | | | | | | | | | | | | | | | | | | | | | | | | | | | | | | | | | | |  |  |  |  |  |  |  |  |
| **□** A surgical airway is performed when endotracheal intubation is difficult.  **□** SGA is not useful as a rescue ventilation device.  **□** Financial problem **□** unfamiliar with SGA | | | | | | | | | | | | | | | | | | | | | | | | | | | | | | | | | | | | | | | | | | | | | | | | | | | | | | | | | | | | | | | | | | | | | | | | | | | | | | | | | | | | | | | | | | | | | | | | | | | | | | | | | | | | |  |  |  |  |  |  |  |  |
| **□** Other reason (Please specify) | | | | | | | | | | | | | | | | | | | | | | | | | | | | | | | | | | | | | | | | |  | | | | | | | | | | | | | | | | | | | | | | | | | | | | | | | | | | | | | | | | | | | | | | | | | | | | | | | | | | | | | | | | | | | |  |  |  |  |  |  |  |  |
| *D.* Oral airway | | | | | | | | | | | | | | | | | | | | | | | | | | | | | | | | | | | | | | | | | **□ Yes □ No** | | | | | | | | | | | | | | | | | | | | | | | | | | | | | | | | | | | | | | | | | | | | | | | | | | | | | | | | | | | | | | | | | | | |  |  |  |  |  |  |  |  |
| *E.* Nasal airway | | | | | | | | | | | | | | | | | | | | | | | | | | | | | | | | | | | | | | | | | **□ Yes □ No** | | | | | | | | | | | | | | | | | | | | | | | | | | | | | | | | | | | | | | | | | | | | | | | | | | | | | | | | | | | | | | | | | | | |  |  |  |  |  |  |  |  |
| (4) Surgical airway device | | | | | | | | | | | | | | | | | | | | | | | | | | | | | | | | | | | | | | | | |  | | | | | |  | | |  | | | | |  | |  | | | |  | | | |  | | | |  | | | |  | | | |  | | | |  | | | |  | | | |  | | | |  | | | |  | | | | |  | | | | | | |  |  |  |  |  |  |  |  |
| *A.* Cricothyroidotomy kit | | | | | | | | | | | | | | | | | | | | | | | | | | | | | | | | | | | | | | | | | **□ Yes □ No**  **□ Only scalpel and hemostat** | | | | | | | | | | | | | | | | | | | | | | | | | | | | | | | | | | | | | | | | | | | | | | | | | | | | | | | | | | | | | | | | | | | |  |  |  |  |  |  |  |  |
| (5) Device to confirm endotracheal intubation | | | | | | | | | | | | | | | | | | | | | | | | | | | | | | | | | | | | | | | | |  | | | | | |  | | |  | | | | |  | |  | | | |  | | | |  | | | |  | | | |  | | | |  | | | |  | | | |  | | | |  | | | |  | | | |  | | | | |  | | | | | | |  |  |  |  |  |  |  |  |
| *A.* Quantitative capnometry (EtCO_2_ monitor) | | | | | | | | | | | | | | | | | | | | | | | | | | | | | | | | | | | | | | | | | **□ Yes □ No** | | | | | | | | | | | | | | | | | | | | | | | | | | | | | | | | | | | | | | | | | | | | | | | | | | | | | | | | | | | | | | | | | | | |  |  |  |  |  |  |  |  |
| *B.* Colorimetric capnometry | | | | | | | | | | | | | | | | | | | | | | | | | | | | | | | | | | | | | | | | | **□ Yes □ No** | | | | | | | | | | | | | | | | | | | | | | | | | | | | | | | | | | | | | | | | | | | | | | | | | | | | | | | | | | | | | | | | | | | |  |  |  |  |  |  |  |  |
| *C.* If you answered ***Yes*** to ***A*** or ***B*** or ***both***, please answer the following question: | | | | | | | | | | | | | | | | | | | | | | | | | | | | | | | | | | | | | | | | | | | | | | | | | | | | | | | | | | | | | | | | | | | | | | | | | | | | | | | | | | | | | | | | | | | | | | | | | | | | | | | | | | | | |  |  |  |  |  |  |  |  |
| Does your ED staff use capnometry to confirm correct tube placement? | | | | | | | | | | | | | | | | | | | | | | | | | | | | | | | | | | | | | | | | | **□ Routinely □ Sometimes**  **□ Never** | | | | | | | | | | | | | | | | | | | | | | | | | | | | | | | | | | | | | | | | | | | | | | | | | | | | | | | | | | | | | | | | | | | |  |  |  |  |  |  |  |  |
| *D.* If you answered yes to ***both A and B***, which device is mainly used? | | | | | | | | | | | | | | | | | | | | | | | | | | | | | | | | | | | | | | | | | **□ Quantitative capnometry**  **□ Colorimetric capnometry** | | | | | | | | | | | | | | | | | | | | | | | | | | | | | | | | | | | | | | | | | | | | | | | | | | | | | | | | | | | | | | | | | | | |  |  |  |  |  |  |  |  |
| *E.* If ***“Routinely”*** was **not** chosen in ***C,*** what are the reasons? (Please select all that apply.) | | | | | | | | | | | | | | | | | | | | | | | | | | | | | | | | | | | | | | | | | | | | | | | | | | | | | | | | | | | | | | | | | | | | | | | | | | | | | | | | | | | | | | | | | | | | | | | | | | | | | | | | | | | | |  |  |  |  |  |  |  |  |
| **□** endotracheal intubation confirmed with other methods (e.g., auscultation, tube fogging, chest rise, direct visualization) **□** shortage of capnometry **□**financial problem  **□** unfamiliar with capnometry **□** capnometry use is not standardized among physicians | | | | | | | | | | | | | | | | | | | | | | | | | | | | | | | | | | | | | | | | | | | | | | | | | | | | | | | | | | | | | | | | | | | | | | | | | | | | | | | | | | | | | | | | | | | | | | | | | | | | | | | | | | | | |  |  |  |  |  |  |  |  |
| **□** Other reason (Please specify) | | | | | | | | | | | | | | | | | | | | | | | | | | | | | | | | | | | | | | | | |  | | | | | | | | | | | | | | | | | | | | | | | | | | | | | | | | | | | | | | | | | | | | | | | | | | | | | | | | | | | | | | | | | | | |  |  |  |  |  |  |  |  |
| (6) Endotracheal intubation facilitating drugs/Reversal | | | | | | | | | | | | | | | | | | | | | | | | | | | | | | | | | | | | | | | | | | | | | | | | | | | | | | | | | | | | | | | | | | | | | | | | | | | | | | | | | | | | | | | | | | | | | | | | | | | | | | | | | | | | |  |  |  |  |  |  |  |  |
| Are the following drugs available in your ED? (Please select all that apply.) | | | | | | | | | | | | | | | | | | | | | | | | | | | | | | | | | | | | | | | | | | | | | | | | | | | | | | | | | | | | | | | | | | | | | | | | | | | | | | | | | | | | | | | | | | | | | | | | | | | | | | | | | | | | |  |  |  |  |  |  |  |  |
| 1. Analgesics | | | | | | | | | | | | | | | | | | | | | | | | | | | | | | | | | | | | | | | | | | | | | | | | | | | | | | | | | | | | | | | | | | | | | | | | | | | | | | | | | | | | | | | | | | | | | | | | | | | | | | | | | | | | |  |  |  |  |  |  |  |  |
| □ Fentanyl □ Morphine □ Remifentanil □ Ketamine □ Pentazocin □ Buprenorphine  □ Tramadol □ Lidocaine | | | | | | | | | | | | | | | | | | | | | | | | | | | | | | | | | | | | | | | | | | | | | | | | | | | | | | | | | | | | | | | | | | | | | | | | | | | | | | | | | | | | | | | | | | | | | | | | | | | | | | | | | | | | |  |  |  |  |  |  |  |  |
| □ Other (Please specify) | | | | | | | | | | | | | | | | | | | | | | | | | | | | | | | | | | |  | | | | | | | | | | | | | | | | | | | | | | | | | | | | | | | | | | | | | | | | | | | | | | | | | | | | | | | | | | | | | | | | | | | | | | | | | |  |  |  |  |  |  |  |  |
| *B.* Sedatives | | | | | | | | | | | | | | | | | | | | | | | | | | | | | | | | | | | | | | | | | | | | | | | | | | | | | | | | | | | | | | | | | | | | | | | | | | | | | | | | | | | | | | | | | | | | | | | | | | | | | | | | | | | | |  |  |  |  |  |  |  |  |
| □ Midazolam □ Diazepam □ Propofol □ Thiopental □ Dexmedetomidine  □ Droperidol □ Haloperidol | | | | | | | | | | | | | | | | | | | | | | | | | | | | | | | | | | | | | | | | | | | | | | | | | | | | | | | | | | | | | | | | | | | | | | | | | | | | | | | | | | | | | | | | | | | | | | | | | | | | | | | | | | | | |  |  |  |  |  |  |  |  |
| □ Other (Please specify) | | | | | | | | | | | | | | | | | | | | | | | | | | | | | | | | | | |  | | | | | | | | | | | | | | | | | | | | | | | | | | | | | | | | | | | | | | | | | | | | | | | | | | | | | | | | | | | | | | | | | | | | | | | | | |  |  |  |  |  |  |  |  |
| *C.* Neuromuscular blocking agents | | | | | | | | | | | | | | | | | | | | | | | | | | | | | | | | | | | | | | | | | | | | | | | | | | | | | | | | | | | | | | | | | | | | | | | | | | | | | | | | | | | | | | | | | | | | | | | | | | | | | | | | | | | | |  |  |  |  |  |  |  |  |
| □ Rocuronium □ Vecuronium □ Pancuronium □ Succinylcholine | | | | | | | | | | | | | | | | | | | | | | | | | | | | | | | | | | | | | | | | | | | | | | | | | | | | | | | | | | | | | | | | | | | | | | | | | | | | | | | | | | | | | | | | | | | | | | | | | | | | | | | | | | | | |  |  |  |  |  |  |  |  |
| □ Other (Please specify) | | | | | | | | | | | | | | | | | | | | | | | | | | | | | | | | | | | | | | | | | | | | | | |  | | | | | | | | | | | | | | | | | | | | | | | | | | | | | | | | | | | | | | | | | | | | | | | | | | | | | | | | | | | | | |  |  |  |  |  |  |  |  |
| *D.* Reversal agents | | | | | | | | | | | | | | | | | | | | | | | | | | | | | | | | | | | | | | | | | | | | | | | | | | | | | | | | | | | | | | | | | | | | | | | | | | | | | | | | | | | | | | | | | | | | | | | | | | | | | | | | | | | | |  |  |  |  |  |  |  |  |
| □ Sugammadex □ Naloxone □ Flumazenil □ Neostigmine | | | | | | | | | | | | | | | | | | | | | | | | | | | | | | | | | | | | | | | | | | | | | | | | | | | | | | | | | | | | | | | | | | | | | | | | | | | | | | | | | | | | | | | | | | | | | | | | | | | | | | | | | | | | |  |  |  |  |  |  |  |  |
| □ Other (Please specify) | | | | | | | | | | | | | | | | | | | | | | | | | | | | | | | | | | | | | | | | |  | | | | | | | | | | | | | | | | | | | | | | | | | | | | | | | | | | | | | | | | | | | | | | | | | | | | | | | | | | | | | | | | | | | |  |  |  |  |  |  |  |  |
| (7) Portable packaged unit containing difficult airway management (DAM) equipment | | | | | | | | | | | | | | | | | | | | | | | | | | | | | | | | | | | | | | | | | | | | | | | | | | | | | | | | | | | | | | | | | | | | | | | | | | | | | | | | | | | | | | | | | | | | | | | | | | | | | | | | | | | | |  |  |  |  |  |  |  |  |
| *A.* Does your ED have a dedicated portable packaged unit (e.g., DAM cart, DAM bag)? | | | | | | | | | | | | | | | | | | | | | | | | | | | | | | | | | | | | | | | | | **□ Yes □ No** | | | | | | | | | | | | | | | | | | | | | | | | | | | | | | | | | | | | | | | | | | | | | | | | | | | | | | | | | | | | | | | | | | | |  |  |  |  |  |  |  |  |
| *B.* If yes, is the following equipment contained in the portable packaged unit?  (Please select all that apply.) | | | | | | | | | | | | | | | | | | | | | | | | | | | | | | | | | | | | | | | | | | | | | | | | | | | | | | | | | | | | | | | | | | | | | | | | | | | | | | | | | | | | | | | | | | | | | | | | | | | | | | | | | | | | |  |  |  |  |  |  |  |  |
| □ Rigid laryngoscope blades in various designs and sizes □ Rigid video laryngoscope  □ Tracheal tubes in assorted sizes □ Magill forceps □ Stylet □ Gum elastic bougie  □ Airway (oral/nasal) □ Supraglottic airway device □ Surgical airway device □ Capnometry  □ Sugammadex □ Bag valve mask □ Yankauer suction tip | | | | | | | | | | | | | | | | | | | | | | | | | | | | | | | | | | | | | | | | | | | | | | | | | | | | | | | | | | | | | | | | | | | | | | | | | | | | | | | | | | | | | | | | | | | | | | | | | | | | | | | | | | | | |  |  |  |  |  |  |  |  |
| □ Other devices (please specify) | | | | | | | | | | | | | | | | | | | | | | | | | | | | | | | |  | | | | | | | | | | | | | | | | | | | | | | | | | | | | | | | | | | | | | | | | | | | | | | | | | | | | | | | | | | | | | | | | | | | | | | | | | | | | |  |  |  |  |  |  |  |  |
| 1. **ED physicians** | | | | | | | | | | | | | | | | | | | | | | | | | | | | | | | | | | | | | | | | | | | | | | | | | | | | | | | | | | | | | | | | | | | | | | | | | | | | | | | | | | | | | | | | | | | | | | | | | | | | | | | | | | | | |  |  |  |  |  |  |  |  |
| *A.* How many ED physicians does your hospital have? Please include senior residents (post-graduate year ≥3), but not junior residents (post-graduate year 1 or 2). | | | | | | | | | | | | | | | | | | | | | | | | | | | | | | | | | | | | | | | | |  | | | | | | | | | | | | | | | | | | | | | | | | | | | | | | | | | | | | | | | | | | | | | | | | | | | | | | | | | | | | | | | | | | | |  |  |  |  |  |  |  |  |
| *B.* How many physicians are usually on duty during the day in your ED? | | | | | | | | | | | | | | | | | | | | | | | | | | | | | | | | | | | | | | | | | **□ Two or more**  **□ One (+ junior residents)** | | | | | | | | | | | | | | | | | | | | | | | | | | | | | | | | | | | | | | | | | | | | | | | | | | | | | | | | | | | | | | | | | | | |  |  |  |  |  |  |  |  |
| *C.* How many physicians are usually on duty overnight in your ED? | | | | | | | | | | | | | | | | | | | | | | | | | | | | | | | | | | | | | | | | | **□ Two or more**  **□ One (+ junior residents)** | | | | | | | | | | | | | | | | | | | | | | | | | | | | | | | | | | | | | | | | | | | | | | | | | | | | | | | | | | | | | | | | | | | |  |  |  |  |  |  |  |  |
| *D.* If you answered ***“One”*** to ***C***, can you obtain in-house experienced (anesthetic or critical care medicine) back-up coverage overnight? | | | | | | | | | | | | | | | | | | | | | | | | | | | | | | | | | | | | | | | | | **□ Yes □ No** | | | | | | | | | | | | | | | | | | | | | | | | | | | | | | | | | | | | | | | | | | | | | | | | | | | | | | | | | | | | | | | | | | | |  |  |  |  |  |  |  |  |
| *E.* Of these ED physicians, how many are board-certified in the fields listed below?*  *Physicians may have more than one board certification. | | | | | | | | | | | | | | | | | | | | | | | | | | | | | | | | | | | | | | | | | | | | | | | | | | | | | | | | | | | | | | | | | | | | | | | | | | | | | | | | | | | | | | | | | | | | | | | | | | | | | | | | | | | | |  |  |  |  |  |  |  |  |
| Emergency medicine | | | | | | | | | | | | | | | | | | | | |  | | | | | | | | | | | | | | | | General surgery | | | | | | | | | | | | | | | | | | | | | | | | | | | | | | | | | | | |  | | | | | | | | | | | | | | | | | | | | | | | | | | | | | | | | | | | |  |  |  |  |  |  |  |  |
| Intensive care | | | | | | | | | | | | | | | | | | | | |  | | | | | | | | | | | | | | | | Anesthesia | | | | | | | | | | | | | | | | | | | | | | | | | | | | | | | | | | | |  | | | | | | | | | | | | | | | | | | | | | | | | | | | | | | | | | | | |  |  |  |  |  |  |  |  |
| Cardiovascular surgery | | | | | | | | | | | | | | | | | | | | |  | | | | | | | | | | | | | | | | Cranial surgery | | | | | | | | | | | | | | | | | | | | | | | | | | | | | | | | | | | |  | | | | | | | | | | | | | | | | | | | | | | | | | | | | | | | | | | | |  |  |  |  |  |  |  |  |
| Orthopedics | | | | | | | | | | | | | | | | | | | | |  | | | | | | | | | | | | | | | | Cardiovascular medicine | | | | | | | | | | | | | | | | | | | | | | | | | | | | | | | | | | | |  | | | | | | | | | | | | | | | | | | | | | | | | | | | | | | | | | | | |  |  |  |  |  |  |  |  |
| Respiratory medicine | | | | | | | | | | | | | | | | | | | | |  | | | | | | | | | | | | | | | | Renal medicine | | | | | | | | | | | | | | | | | | | | | | | | | | | | | | | | | | | |  | | | | | | | | | | | | | | | | | | | | | | | | | | | | | | | | | | | |  |  |  |  |  |  |  |  |
| Pediatrics | | | | | | | | | | | | | | | | | | | | |  | | | | | | | | | | | | | | | | Other board certifications | | | | | | | | | | | | | | | | | | | | | | | | | | | | | | | | | | | |  | | | | | | | | | | | | | | | | | | | | | | | | | | | | | | | | | | | |  |  |  |  |  |  |  |  |
| 1. **Airway management teaching program** | | | | | | | | | | | | | | | | | | | | | | | | | | | | | | | | | | | | | | | | | | | | | | | | | | | | | | | | | | | | | | | | | | | | | | | | | | | | | | | | | | | | | | | | | | | | | | | | | | | | | | | | | | | | |  |  |  |  |  |  |  |  |
| Are any of the following airway teaching programs for senior residents available in your ED?  (Please select all that apply.) | | | | | | | | | | | | | | | | | | | | | | | | | | | | | | | | | | | | | | | | | | | | | | | | | | | | | | | | | | | | | | | | | | | | | | | | | | | | | | | | | | | | | | | | | | | | | | | | | | | | | | | | | | | | |  |  |  |  |  |  |  |  |
| □ Anesthesiology rotation (training in the hospital operating room) □DAM simulation training  □ Didactic DAM lecture □ surgical airway training using a simulator, animal model, or a cadaver | | | | | | | | | | | | | | | | | | | | | | | | | | | | | | | | | | | | | | | | | | | | | | | | | | | | | | | | | | | | | | | | | | | | | | | | | | | | | | | | | | | | | | | | | | | | | | | | | | | | | | | | | | | | |  |  |  |  |  |  |  |  |
| □ Other training program (please specify) | | | | | | | | | | | | | | | | | | | | | | | | | | | | | | | | | | | | | | | | |  | | | | | | | | | | | | | | | | | | | | | | | | | | | | | | | | | | | | | | | | | | | | | | | | | | | | | | | | | | | | | | | | | | | |  |  |  |  |  |  |  |  |
| 1. **Free comments** | | | | | | | | | | | | | | | | | | | | | | | | | | | | | | | | | | | | | | | | | | | | | | | | | | | | | | | | | | | | | | | | | | | | | | | | | | | | | | | | | | | | | | | | | | | | | | | | | | | | | | | | | | | | |  |  |  |  |  |  |  |  |
| 1. Are there any recommendations or protocols in place when you perform emergency endotracheal intubation in your ED? | | | | | | | | | | | | | | | | | | | | | | | | | | | | | | | | | | | | | | | | | | | | | | | | | | | | | | | | | | | | | | | | | | | | | | | | | | | | | | | | | | | | | | | | | | | | | | | | | | | | | | | | | | | | |  |  |  |  |  |  |  |  |
|  | | | | | | | | | | | | | | | | | | | | | | | | | | | | | | | | | | | | | | | | | | | | | | | | | | | | | | | | | | | | | | | | | | | | | | | | | | | | | | | | | | | | | | | | | | | | | | | | | | | | | | | | | | | | |  |  |  |  |  |  |  |  |
| 1. Other comments | | | | | | | | | | | | | | | | | | | | | | | | | | | | | | | | | | | | | | | | | | | | | | | | | | | | | | | | | | | | | | | | | | | | | | | | | | | | | | | | | | | | | | | | | | | | | | | | | | | | | | | | | | | | |  |  |  |  |  |  |  |  |
|  | | | | | | | | | | | | | | | | | | | | | | | | | | | | | | | | | | | | | | | | | | | | | | | | | | | | | | | | | | | | | | | | | | | | | | | | | | | | | | | | | | | | | | | | | | | | | | | | | | | | | | | | | | | | |  |  |  |  |  |  |  |  |
|  | |  | |  | |  | |  | |  | |  | |  | |  | |  | |  | | |  | |  | |  | |  | |  | | |  | | | | | | |  | |  | |  | | | | | |  | | |  | | | | |  | | | |  | | | |  | | | |  | | | |  | | | |  | | | |  | | | |  | | | |  | | | |  | | | | |  | | | | |  | | |  | | | |  | |  | |  |
| **Thank you very much for your time and collaboration.** | | | | | | | | | | | | | | | | | | | | | | | | | | | | | | | | | | | | | | | | | | | | | | | | | | | | | | | | | | | | | | | | | | | | | | | | | | | | | | | | | | | | | | | | | | | | | | | | | | | | | | | | | | | | |  |  |  |  |  |  |  |  |
