# Supplementary material for: Human and equipment resources for difficult airway management, airway education programs, and capnometry use in Japanese emergency departments: a nationwide cross-sectional study
Source: Int J Emerg Med. 2017 Sep 13;10:28. doi: 10.1186/s12245-017-0155-6 (PMC5597568; doi:10.1186/s12245-017-0155-6)
Supplement: Supplementary file 2 — International comparison of outcomes of interests with the outcome determined in this study. (DOCX 14 kb) [file 12245_2017_155_MOESM2_ESM.docx]

| Reference | Country | Type of EDs | 24-h back-up coverage (%) | SGA (%) | DAM cart (%) | Surgical airway device (%) | Neuromuscular blocking agents (%) | Anesthesia rotation (%) | Routine use of capnometry for ETI (%) |
| --- | --- | --- | --- | --- | --- | --- | --- | --- | --- |
| Levitan et al. [29], 1999 | USA | Mixed | N/R | 52.6 | N/R | 67.3^a^ | N/R | N/R | N/R |
| Morton et al. [28], 2000 | England | Adult | N/R | 83.8 | N/R | 98 | N/R | N/R | N/R |
| Walsh et al. [30], 2004 | Ireland | Mixed | N/R | 100 | 33.3 | 100 | N/R | N/R | N/R |
| Deiorio et al. [31], 2005 | USA | Mixed | N/R | N/R | N/R | N/R | N/R | N/R | 14^b^ and 40^c^ |
| Reeder et al. [36], 2005 | USA | Mixed | N/R | 66.1 | N/R | 94.9 | N/R | 86.4 | N/R |
| Langhan et al. [34], 2008 | USA | Pediatric | N/R | N/R | N/R | N/R | N/R | N/R | 69 |
| Losek et al. [35], 2008 | USA | Pediatric | N/R | 90.2 | N/R | 82 | N/R | 43 | N/R |
| Browne et al. [33], 2015 | New Zealand | Mixed | N/R | N/R | N/R | N/R | N/R | 53.3 | N/R |
| Present study, 2016 | Japan | Mixed | 69.4 | 51.5 | 49.7 | 95.7 | 68.5 | 38.6 | 47.8 |

**Additional file 2: Table S1.** International comparison of outcomes of interests with the outcome determined in this study

*DAM* difficult airway management, *ED* emergency department, *ETI* endotracheal intubation, *N/R* not recorded, *SGA* supraglottic airway device

^a^ Indicates the availability of a transtracheal jet ventilation system.

^b^ Part I of the study. 19 of 138 randomly surveyed ED physicians

^c^ Part II of the study. 6 of 15 selected institutions committed to monitoring airway practices.
