# Supplementary material for: Human and equipment resources for difficult airway management, airway education programs, and capnometry use in Japanese emergency departments: a nationwide cross-sectional study
Source: Int J Emerg Med. 2017 Sep 13;10:28. doi: 10.1186/s12245-017-0155-6 (PMC5597568; doi:10.1186/s12245-017-0155-6)
Supplement: Supplementary file 3 — Characteristic differences between respondent vs. non-respondent emergency departments (EDs). (DOCX 13 kb) [file 12245_2017_155_MOESM3_ESM.docx]

| **ED type** | **Respondent EDs (N=324)** | **Non-respondent EDs (N=206)** | ***p*** |
| --- | --- | --- | --- |
| Academic^a^ | 79 (24.4) | 28 (13.6) | 0.003 |
| High-volume^bc^ | 80 (25.1) | Not available | Not available |
| Tertiary^d^ | 184 (56.8) | 81 (39.3) | <0.001 |
| Urban^e^ | 117 (36.1) | 68 (33.0) | 0.5 |
| Pediatric^f^ | 8 (2.5) | 4 (1.9) | 0.8 |

**Additional file 3: Table S2. Characteristic differences between respondent vs. non-respondent emergency departments (EDs)**

Data are presented as n (%).

^a^Defined as EDs in university-affiliated hospitals.

^b^The number of respondent EDs was 319 because of 5 missing data.

^c^Defined as EDs in the upper quartile of annual ambulance visits (>5728).

^d^Defined as EDs in referral medical centers of regional emergency medical control that are certified by the Japanese Ministry of Health, Labour and Welfare.

^e^Defined using the census grouping criteria by the Statistics Bureau of the Japanese Ministry of Internal Affairs and Communications.

^f^Defined as EDs with a referral resource for critically ill children of communities in nearby regions that are certified by the Japanese Ministry of Health, Labour and Welfare.
